# Identification of Transcription Factor Genes and Functional Characterization of *PlMYB1* From *Pueraria lobata*

**DOI:** 10.3389/fpls.2021.743518

**Published:** 2021-10-08

**Authors:** Guoan Shen, Ranran Wu, Yaying Xia, Yongzhen Pang

**Affiliations:** ^1^The Institute of Medicinal Plant Development, Chinese Academy of Medical Sciences, Peking Union Medical College, Beijing, China; ^2^Key Laboratory of Plant Resources/Beijing Botanical Garden, Institute of Botany, Chinese Academy of Sciences, Beijing, China; ^3^Institute of Animal Sciences, Chinese Academy of Agricultural Sciences, Beijing, China

**Keywords:** *Pueraria lobata*, transcriptome sequencing, transcription factors, MYB, isoflavonoid biosynthesis

## Abstract

Kudzu, *Pueraria lobata*, is a traditional Chinese food and medicinal herb that has been commonly used since ancient times. Kudzu roots are rich sources of isoflavonoids, e.g., puerarin, with beneficial effects on human health. To gain global information on the isoflavonoid biosynthetic regulation network in kudzu, *de novo* transcriptome sequencings were performed using two genotypes of kudzu with and without puerarin accumulation in roots. RNAseq data showed that the genes of the isoflavonoid biosynthetic pathway were significantly represented in the upregulated genes in the kudzu with puerarin. To discover regulatory genes, 105, 112, and 143 genes encoding MYB, bHLH, and WD40 transcription regulators were identified and classified, respectively. Among them, three *MYB*, four *bHLHs*, and one *WD40* gene were found to be highly identical to their orthologs involved in flavonoid biosynthesis in other plants. Notably, the expression profiles of *PlMYB1*, *PlHLH3-4*, and *PlWD40-1* genes were closely correlated with isoflavonoid accumulation profiles in different tissues and cell cultures of kudzu. Over-expression of *PlMYB1* in *Arabidopsis thaliana* significantly increased the accumulation of anthocyanins in leaves and proanthocyanidins in seeds, by activating *AtDFR*, *AtANR*, and *AtANS* genes. Our study provided valuable comparative transcriptome information for further identification of regulatory or structural genes involved in the isoflavonoid pathway in *P. lobata*, as well as for bioengineering of bioactive isoflavonoid compounds.

## Introduction

*Pueraria lobata*, commonly known as kudzu, belongs to the *Leguminosae* family. The roots of kudzu are enriched in starch, which has traditionally been used as a source of food consumption and beverage production in East Asia. Additionally, kudzu has been used for centuries in Chinese traditional medicine as an antipyretic, antidiarrheic, and antiemetic agent (Keung and Vallee, 1998; Wong et al., 2011). Kudzu roots are rich resources of natural product isoflavonoids, including daidzein, genistein, formononetin, and puerarin (also called daidzein 8-*C*-glycoside) (Prasain et al., 2003; Li et al., 2014). Among these isoflavonoids, puerarin is the major effective ingredient with antioxidative, antidiabetic, and antithrombotic effects (Hien et al., 2010; Liu et al., 2013), and it could also help to cure non-alcoholic fatty liver diseases, alcohol-induced adipogenesis, and osteonecrosis (Xia et al., 2013). The beneficial therapeutic effects of isoflavonoids, in particular puerarin, have made *P. lobata* an interesting plant species in investigating isoflavonoid biosynthesis and regulation.

Isoflavonoids are almost exclusively limited to the family of *Leguminosae*, the biosynthesis of which share the common upstream pathway with flavonoids (Supplementary Figure 1). Three molecules of malony-CoA were condensed with one molecule of 4-coumaroyl-CoA to form naringenin-chalcone or isoliquiritigenin, under the catalysis of chalcone synthase (CHS) or chalcone synthase/chalcone reductase (CHR). Chalcone isomerase (CHI) then catalyzes the following reaction to form naringenin and liquiritigenin, respectively. Subsequently, these two products were converted to daizein and genistein by the sequential actions of isoflavone synthase (IFS) and hydroxyflavanone dehydratase (HID). Finally, UDP-glycosyltransferase (UGT) could add glucose moiety to these two aglycone intermediates to form different *O*-glucosides at C7 or C5 position (He et al., 2011). Puerarin has been revealed to be synthesized *via* daidzein (He et al., 2011; Li et al., 2014; Wang et al., 2017). Although several structural genes, such as *4-Coumarate: Coenzyme A* ligases, and *UGTs* involved in the isoflavonoid pathway have been identified in kudzu (He et al., 2011; Li et al., 2014; Wang et al., 2017), transcription factors were less identified for the regulation of isoflavonoid biosynthesis in kudzu.

Transcriptional regulation of flavonoid pathway has been extensively studied in many plant species, such as *Zea mays* (Grotewold et al., 1998; Carey et al., 2004), *Arabidopsis thaliana* (Nesi et al., 2000, 2001; Zhu et al., 2009; Wada et al., 2014; Shin et al., 2015), *Malus domestica* (Espley et al., 2007), and *Vitis vinifera* (Terrier et al., 2009; Hichri et al., 2010; Ali et al., 2011; Huang et al., 2014). These studies have established the key roles of transcription factors in the regulation of most structural genes. Up to now, at least six distinct types of transcription regulators, which are MYB, bHLH, WD40, WRKY, Zinc finger, and MADS-box proteins, have been proven to be involved in the regulation of secondary metabolite biosynthetic pathway (Sun et al., 2019; Deng et al., 2020a, b; Hao et al., 2020, 2021; Anguraj Vadivel et al., 2021; Fan et al., 2021; Liu et al., 2021; Mao et al., 2021; Zhou et al., 2021a, b). Among them, transcription regulators of MYB, bHLH, and WD40 function individually or collaborate with each other as MBW complex to control multiple enzymatic steps in the flavonoid pathway (Ramsay and Glover, 2005; Gonzalez et al., 2008; Xu et al., 2014).

MYB proteins, R2R3-MYBs in particular, are major players as the positive or negative regulators toward key biosynthetic genes required for the production of flavonoids (Jiu et al., 2021; Li et al., 2021; Shi et al., 2021). In *Arabidopsis*, flavonoid biosynthesis is mainly regulated by a set of R2R3-MYB transcription factors. Transparent Testa 2 (AtTT2), Production of Anthocyanin Pigments 1 (AtPAP1), and AtMYB11 could activate flavonoid biosynthesis, whereas AtMYBL2 and others repressed the biosynthesis of flavonoids (Xu et al., 2014).

The first bHLH (the basic helix-loop-helix) protein Lc was reported from maize, which cooperates with the MYB transcription factors C1 and PL1 to regulate the anthocyanin biosynthetic pathway in maize (Ludwig et al., 1989). Several corresponding orthologs of the *Lc* gene have been identified in other plant species (Nesi et al., 2000; Ramsay et al., 2003; Park et al., 2007; Montefiori et al., 2015). The bHLH type protein AtTT8 from *Arabidopsis* is required for normal expression of late flavonoid biosynthetic genes in the siliques of *Arabidopsis* (Nesi et al., 2000), which interacts directly with MYB transcription factors for flavonoid biosynthesis.

WD40 repeat domain proteins are not considered to be real transcription factors in regulating flavonoid pathway, in contrast, they act more likely as a docking platform or receptor for recruiting other proteins (van Nocker and Ludwig, 2003; Zhao et al., 2013). In *Arabidopsis*, the WD40 protein Transparent Testa Glabra 1 (AtTTG1) acts primarily *via* interplaying with AtTT8 partner in the TT2-TT8-TTG1 complex regulating the expression of *AtANR* gene (Nesi et al., 2001; Baudry et al., 2004). Several genes encoding WD40 proteins involved in the regulation of flavonoid pathway have been identified from several plant species, including *AN11* from *Petunia × hybrid* (de Vetten et al., 1997), *PFWD* from *Perilla frutescens* (Sompornpailin et al., 2002), *ZmPAC1* from maize (Carey et al., 2004), *MtWD40-1* from *Medicago truncatula* (Pang et al., 2009), and *WDR1* and *WDR2* from grapevine (Matus et al., 2010).

The biosynthesis of flavonoids/isoflavonoids has been extensively studied in model plants, but it was left behind in non-model plants due to the lack of genomic and genetic information. The current fast-growing RNA-Seq sequencing technique makes it possible to take the advantage of global gene expression data in some species without available genomic information. In this study, we integrated comparative transcriptome information from two kudzu genotypes with contrasting isoflavonoid concentrations in roots and identified a set of putative genes that are possibly involved in isoflavonoid production in kudzu roots, with an emphasis on transcription factors (MYB, bHLH, and WD40). Moreover, our investigation demonstrated that *PlMYB1* was a critical transcription factor involved in the isoflavonoid pathway in *P. lobata*. These findings, thus, provided insights into the regulation network of isoflavonoid biosynthesis in kudzu and offered an important target for bioengineering of bioactive isoflavonoids with beneficial effects on human health.

## Results

### Transcriptome Sequencing and Gene Annotation

**FIGURE 1:**
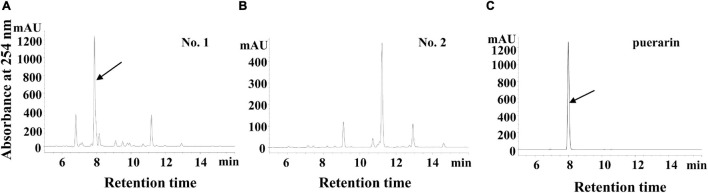
The high-performance liquid chromatography (HPLC) of flavonoid profiles in two types of kudzu plants. **(A)** HPLC chromatogram of the flavonoids in No.1 kudzu plant, the arrowhead indicates puerarin. **(B)** HPLC chromatogram of the flavonoids in No.2 kudzu plant. **(C)** HPLC chromatogram of the puerarin authentic standard.

In a previous report, it was revealed that a kudzu genotype (No. 1, Figure 1A) accumulates a massive amount of puerarin in the roots, whereas the other genotype did not (No. 2, Figure 1B; He et al., 2011). In the present study, we further carried out transcriptome sequencing using the roots of these two types of kudzu plants, attempting to obtain global transcriptome information and to discover new genes involved in the regulation of isoflavonoid biosynthesis by transcriptome comparison.

After sequence cleaning and assembling by using Trinity (trinityrnaseq_r2013-02-25) program (Grabherr et al., 2011), a total of 88,398 unigenes were obtained from these two kudzu plants, and the number of unigenes decreased with the length ranging from short (201 bp) to long (more than 3 kb) (Supplementary Figure 2 and Supplementary Table 1). Among them, 27,515 and 25,175 unigenes were detected solely in Nos. 1 and 2 kudzu, respectively. For the genes expressed in both samples, the reads per kilobases per million reads (RPKM) value of 22, 389 unigenes changed less than twofold between the samples, whereas 5,938 and 7,373 unigenes increased or decreased significantly more than twofold in roots of No. 1 kudzu than in No. 2 kudzu (Supplementary Table 1).

**FIGURE 2:**
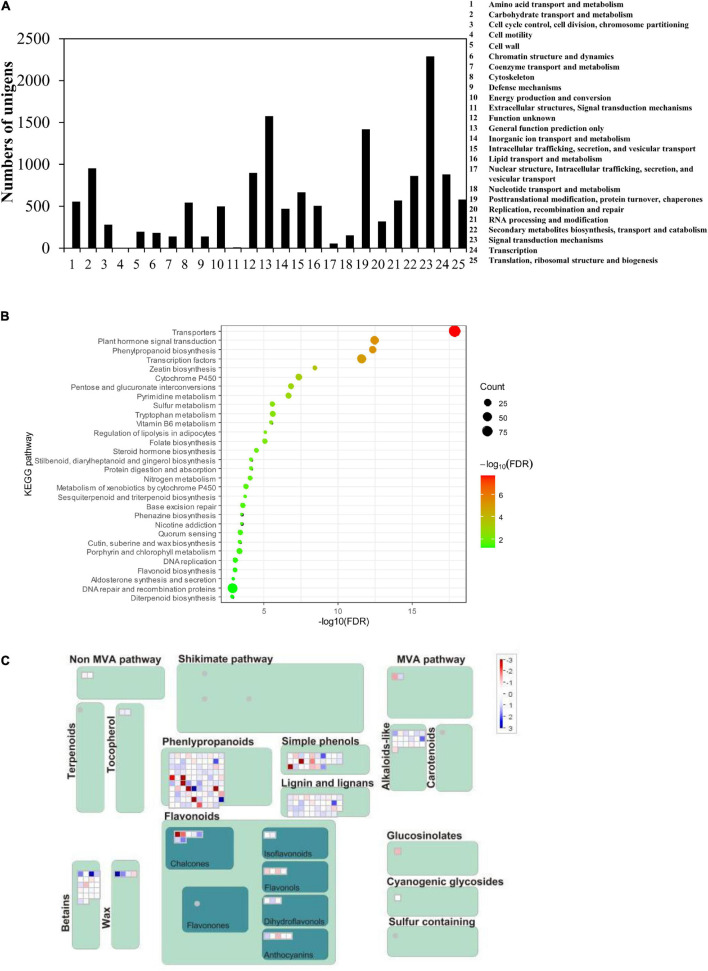
Statistical analysis of the kudzu root transcriptome data. **(A)** Cluster of Orthologous Groups (COG) classification of unigenes of *P. lobata*. A total of 14,896 significantly upregulated unigenes were assigned to 25 classifications. **(B)** Kyoto Encyclopedia of Genes and Genomes (KEGG) enriched pathway highlighting the significantly upregulated genes in the secondary metabolic pathway in the two kudzu transcriptome. **(C)** MapMan overview maps related to flavonoid and phenylpropanoid pathways show evident differences in transcript levels between two kudzu plants. Red indicated upregulated genes and blue indicated downregulated genes.

The function of 14,896 significantly upregulated unigenes in roots of the No. 1 sample was further annotated and classed into 25 groups based on the Cluster of Orthologous Groups of proteins (COG) database. Among them, the highest amounts (2,289) are those having signal transduction mechanisms (Figure 2A). Overall, 863 unigenes were predicted to be involved in the biosynthesis, transport, and catabolism of secondary metabolites (Figure 2A). Among them, 20 unigenes encoding enzymes were related to the isoflavonoid pathway, including 1 *IFS*, 14 *CHS*, 2 *CHR*, and 3 *CHI* genes (Supplementary Table 2).

In particular, KEGG pathway analyses showed that these upregulated genes were significantly enriched in the phenylpropanoid pathway and flavonoid pathway (Figures 2B,C). It was speculated that the accumulation of a higher amount of isoflavonoids resulted from the higher expression of entire pathway genes, like *CHS*, *CHI*, *IFS*, and so on, which should be coordinately regulated by some unknown transcription factors. Therefore, this study mainly focused on the discovery of transcription factor genes, specially *MYB*, *bHLH*, and *WD40* genes.

### Identification and Classification of *MYBs* in the Isoflavonoid Biosynthetic Pathway

In this study, 105 *MYB* genes encoding transcription factors from kudzu roots were identified and confirmed by their conserved domains (Supplementary Table 3). These putative MYB transcription factors were classified into seven different super-families, including DNA_pol_phi superfamily, GAT_SF superfamily, H15 superfamily, Myb_CC_LHEQLE superfamily, SANT superfamily, SKIP_SNW superfamily, and VHS_ENTH_ANTH superfamily (Supplementary Table 3). Among them, the SANT superfamily is the largest superfamily with 65 unigenes (Supplementary Table 3).

R2R3-MYB transcription factors of the SANT superfamily in *Arabidopsis* were divided into 25 different subgroups, and the members from Sg4, Sg5, Sg6, Sg7, and Sg15 subgroups were reported to be involved in the regulation of anthocyanin and proanthocyanidin biosynthesis (Stracke et al., 2001; Hichri et al., 2011). In kudzu, two *MYB* genes *PlMYB4-1* (comp739_c0_seq1) and *PlMYB4-2* (comp41186_c0_seq1) were classed in the Sg4 subgroup, and they shared 34% identity with *AtMYB32* and 88% identity with *AtMYB4*, respectively. The available transcript of *PlMYB4-1* (204 bp) and *PlMYB4-2* (1,110 bp) were predicted to encode a truncated peptide and a full-length protein of 370 amino acid residues, respectively.

Additionally, among *MYB* unigenes predicted in kudzu, *PlMYB1* (comp36832_c0_seq1_3, Genbank accession No. KR698796) showed the highest identity with *GmMYB176* (68%) and *AtTT2* (63%) that were key regulators of isoflavonoid and proanthocyanidin biosynthesis in soybean and *Arabidopsis*, respectively (Nesi et al., 2001; Yi et al., 2010). Moreover, *PlMYB1* (657 bp) was predicted to be full-length and it encodes a deduced protein comprising 219 amino acid residues.

**FIGURE 3:**
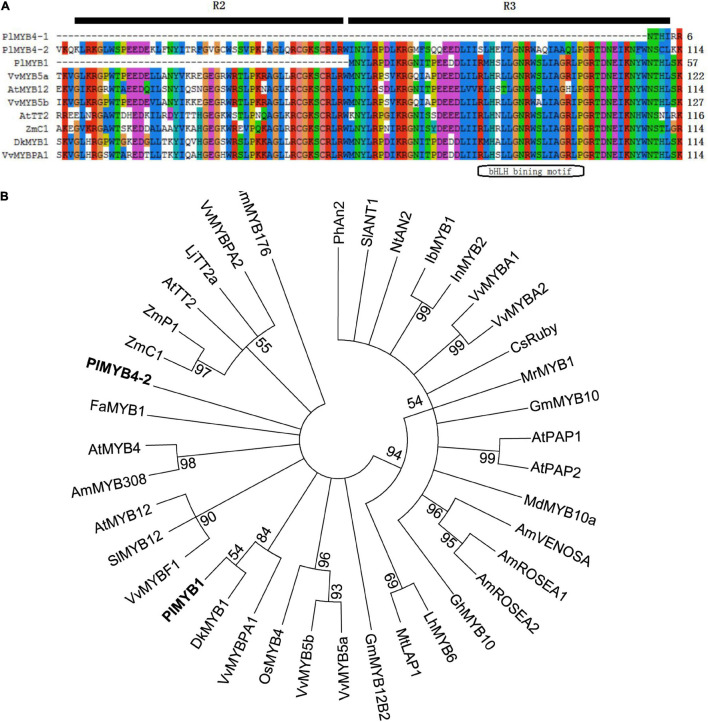
Sequence analysis of MYBs regulating flavonoid biosynthesis at amino acid level. **(A)** Alignment of several MYBs regulating flavonoid biosynthesis. **(B)** Phylogenetic analysis of MYB transcription factors regulating flavonoid metabolism. All the sequences used in the figure are retrieved from the GenBank database, the accession number are as followed (shown in parenthesis): *Antirrhinum majus* AmROSEA1 (ABB83826); AmROSEA2 (ABB83827); AmVENOSA (ABB83828); AmMYB308 (P81393); *Arabidopsis thaliana* AtPAP1 (AAG42001); AtPAP2 (AAG42002); AtTT2 (NP_198405); AtMYB12 (ABB03913); AtMYB4 (NP_195574); *Citrus sinensis* CsRuby (AFB73913); *Diospyros kaki* DkMYB4 (BAI49721); *Fragaria* × *ananassa* FaMYB1 (AAK84064); *Garcinia mangostana* GmMYB10 (ACM62751); *Gerbera hybrid* GhMYB10 (CAD87010); *Ipomoea batatas* IbMYB1 (BAF45114); *Ipomoea nil* InMYB2 (BAE94709); *Solanum lycopersicum* SlANT1 (AAQ55181); *Solanum lycopersicum* SlMYB12 (ACB46530); *Lilium hybrid* LhMYB6 (BAJ05399); *Lotus japonicus* TT2a (BAG12893); *Malus* × *domestica* MdMYB10a (ABB84753); *Medicago truncatula* MtLAP1(ACN79541); *Morella rubra* MrMYB1 (ADG21957); *Nicotiana tabacum* NtAN2 (ACO52470); *Oryza sativa* OsMYB4 (BAA23340); *Petunia* × *hybrida* PhAn2 (AAF66727); *Vitis vinifera* VvMYBA1 (BAD18977); VvMYBA2 (BAD18978); VvMYBPA1 (CAJ90831); VvMYBPA2 (ACK56131); VvMYBF1 (ACV81697); VvMYB5a (AAS68190); VvMYB5b (AAX51291); *Zea mays* ZmC1 (AAA33482); ZmPl (AAA19819); *Glycine max* GmMYB176 (NP_001236048); GmMYB12B2 (AEC13303); *P. lobata* PlMYB1 (AKR04122).

Sequence alignments of the deduced PlMYB1, PlMYB4-1, and PlMYB4-2 proteins showed that the R3 regions were highly conserved compared to other known MYB proteins involved in flavonoid pathway in other species, in particular in the bHLH binding motif (Figure 3A). It is obvious that PlMYB4-2 belonged to the R2R3 group, while PlMYB1 belonged to the R3 group. Phylogenetic analysis showed that PlMYB4-2 was clustered into a group with AtMYB4, FaMYB1, and AmMYB308 that were involved in the general flavonoid pathway, while PlMYB1 was grouped with DkMYB1 and VvMYBPA1 that were involved in the proanthocyanidin biosynthesis (Figure 3B). However, the R2R3 region was missing in PlMYB4-1, which was not pursued further in the current investigation.

### Identification and Classification of *bHLH* Genes in the Isoflavonoid Biosynthetic Pathway in Kudzu

A total of 111 *bHLH* unigenes were predicted in the transcriptome of kudzu roots, and they were classified into corresponding subgroups by sequence identity comparison with orthologs in *Arabidopsis* (Supplementary Table 4). In particular, four kudzu bHLH proteins were grouped into Subgroup III, the ortholog of which was involved in flavonoid biosynthesis in *Arabidopsis* (Heim et al., 2003). *PlbHLH3-1* (Comp36398_c2_seq1, 201 bp) encoding protein shared the highest identity at amino acid level with AtbHLH93 (62%) that belongs to Subgroup IIIb. The other three were assigned to Subgroup IIId. Among them, both *PlbHLH3-2* (comp37021_c0_seq1, 474 bp) and *PlbHLH3-3* (comp37021_c1_seq1, 1,332 bp) shared 63% and 45% identity with *AtbHLH13*, respectively. *PlbHLH3-4* (comp45038_c0_seq1, 1,539 bp, Genbank Accession No. KT236099) shared the highest identity (48%) with *AtbHLH3*.

**FIGURE 4:**
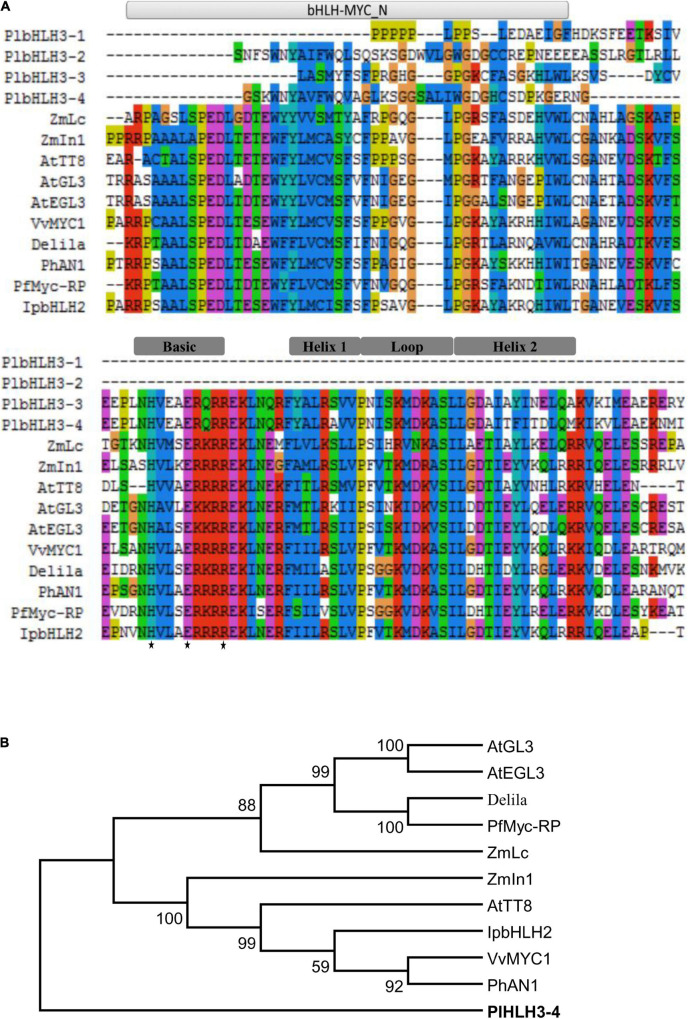
Analysis of bHLHs regulating flavonoid biosynthesis at the amino acid level. **(A)** Alignment of bHLHs regulating flavonoid biosynthesis. **(B)** Phylogenetic analysis of bHLH transcription factors regulating flavonoid metabolism. All the sequences used in the figure are derived from the GenBank database, the accession number are as followed (shown in parenthesis): *Zea mays* ZmLc (NP_001105339); ZmIn1 (AAB03841); *Arabidopsis thaliana* AtTT8 (Q9FT81); AtGL3 (AED94664); AtEGL3 (Q9CAD0); Delila (AAA32663); *Vitis vinifera* VvMYC1 (ACC68685); *Petunia × hybrid* PhAN1 (AAG25928), PhMyc-rp (BAA75513); *Ipomoea purpurea* IpbHLH2 (ABW69688), and *P. lobata* PlbHLH3-4 (AKR04123). The His5-Glu9-Arg13 (H-E-R) motif is indicated with a black star.

Sequence alignments of the deduced PlbHLH3-1, PlbHLH3-2, PlbHLH3-3, and PlbHLH3-4 proteins with other representative bHLH proteins involved in the flavonoid pathway showed that only PlbHLH3-4 had the intact bHLH-MYC_N region (Figure 4A), which is usually present in the N-terminal of bHLH transcription factors regulating phenylpropanoid biosynthesis (Marchler-Bauer et al., 2015). The bHLH-MYC_N domain commonly has the specific DNA-binding ability attributed to the amphipathic affinity of its N-terminus. This was especially apparent in the critical His-Glu-Arg (H-E-R) residues located at positions 5, 9, and 13 in the basic region for PlbHLH3-3 and PlbHLH3-4 (Figure 4A), which could bind to DNA target as reported previously (Atchley and Fitch, 1997; Massari and Murre, 2000; Toledo-Ortiz et al., 2003). The bHLH-MYC_N region was composed of two hydrophobic α-helices linked by a divergent loop, but only PlbHLH3-3 and PlbHLH3-4 contained an intact domain in this region (Figure 4A).

The phylogenic relationship showed that PlbHLH3-3 and PlbHLH3-4 were separated from the other bHLH transcription factors regulating the anthocyanin pathway, such as AtTT8 from *Arabidopsis* and Lc from maize (Figure 4B), suggesting that they are likely involved in other flavonoid branch pathways, e.g., isoflavonoid pathway.

### Identification and Classification of Unigenes Encoding WD40 Repeat Domain Proteins in Kudzu

**FIGURE 5:**
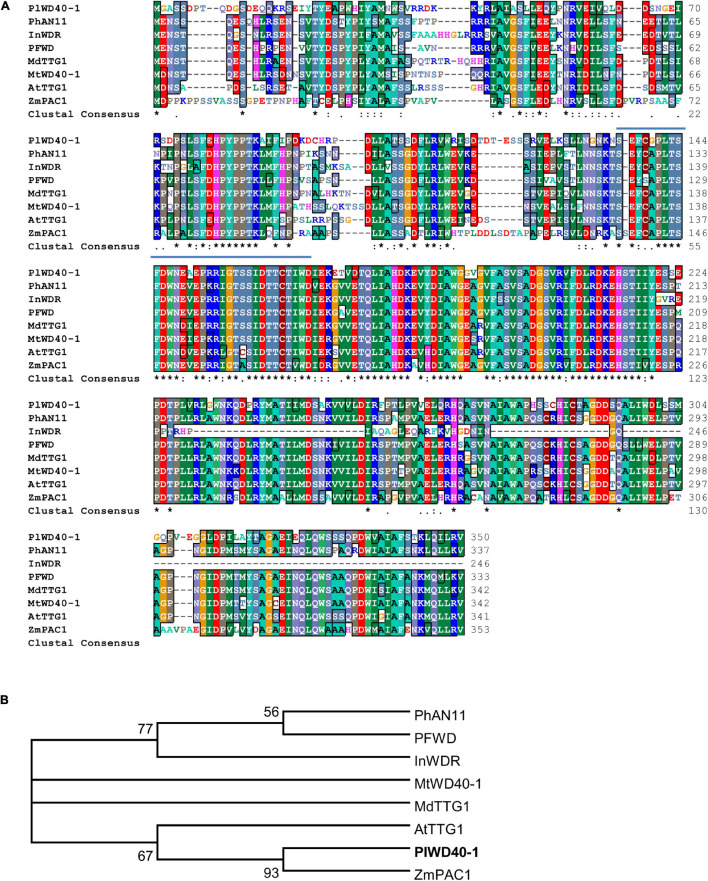
Analysis of PlWD40-1 proteins with WD40 proteins of other plant species at the amino acid level. **(A)** Alignment of WD40s regulating flavonoid biosynthesis. The typical WD domains were indicated in solid lines, and the last two amino acids of each repeat domain are marked with red stars. **(B)** Phylogenetic analysis of WD40 transcription factors regulating flavonoid metabolism. All the sequences used in the figure are derived from the GenBank database; the accession number is as followed (shown in parenthesis): *Arabidopsis thaliana* AtTTG1 (CAB45372); *Malus domestica* MdTTG1 (ADI58760); *Petunia × hybrid* PhAN11 (AAC18914); *Ipomoea nil* InWDR (BAE94407); *Perilla frutescens* PFWD (BAB58883); *Zea mays* ZmPAC1 (AAM76742) and *Medicago truncatula* MtWD40-1 (ABW08112), and *P. lobata* PlWD40-1 (AKR04124.1).

The repeat proteins WD40 are key components in the MBW complex, therefore, we searched and identified a total of 143 unigenes encoding WD40 repeat domain proteins in the transcriptome of kudzu roots (Supplementary Table 5). They were grouped into 16 different super-families, and the WD40 super-family was the largest super-family (96 members, Supplementary Table 5). Among all these predicted WD40 repeat domain proteins, the deduced amino acid sequence of PlWD40-1 (comp42449_c1_seq1_24, Genbank Accession No. AKR04124.1) showed the highest identity (64%) to Transparent testa glabra 1 (AtTTG1) from *Arabidopsis*, 62% to *MtWD40-1* from *M. truncatula*, and 61% to PhAN1 from *P. hybrid*. The WD40 repeat domains are highly conserved among these WD40 proteins (Figure 5A). Phylogenetic analysis showed that PlWD40-1 is most closely related to ZmPAC1 that regulates anthocyanins production in *Zea mays* (Figure 5B). As these WD40 orthologs are involved in the flavonoid pathway, the high identity and close phylogenetic relationship of PlWD40-1 with them suggested that PlWD40-1 may have a similar function in the isoflavonoid pathway in kudzu.

### Expression of Key Transcription Factor Genes Was Closely Correlated With Total Flavonoid Accumulation in Kudzu

**FIGURE 6:**
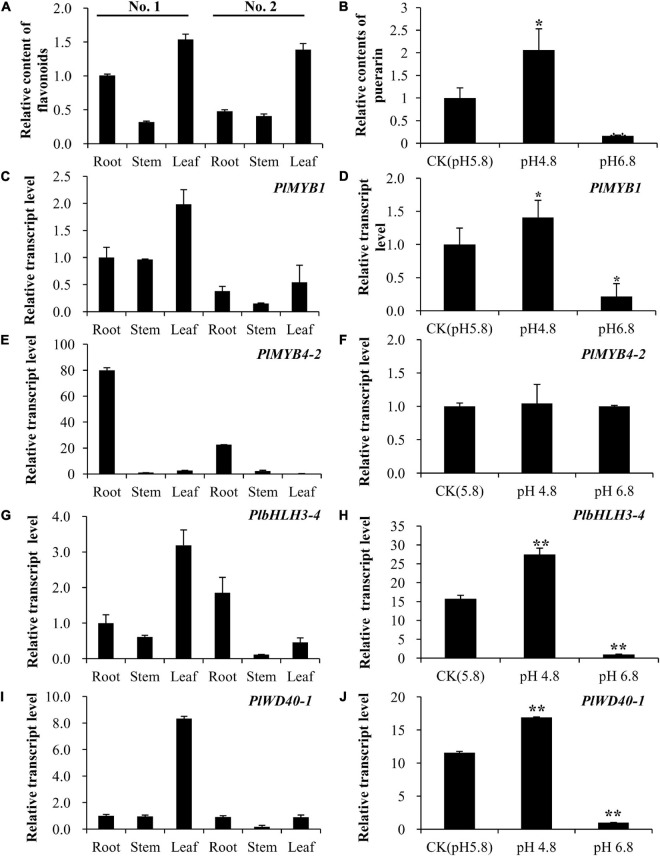
Association analyses of flavonoid accumulation and expression levels of key transcription factor genes in various tissues and cell culture under different pH conditions. **(A)** Accumulation of flavonoids in roots, stems, and leaves of two types of kudzu plants. **(B)** Accumulation of puerarin in cell cultures under different pH conditions. **(C,E,G,I)** The relative transcript levels of *PlMYB1* **(C)**, *PlMYB4-2* **(E)**, *PlbHLH3-4* **(G)**, and *PlWD40-1* **(I)** in roots, stems, and leaves of two types of kudzu plants. **(D,F,H,J)** The relative transcript levels of kudzu *PlMYB1* **(D)**, *PlMYB4-2* **(F)**, *PlbHLH3-4* **(H)**, and *PlWD40-1* **(J)** in cell cultures under pH 4.8, pH 5.8, and pH 6.8.

To further screen candidate transcription factors that play key roles in the isoflavonoid pathway, the association between flavonoid accumulation level and the expression level of candidate genes was investigated. We found that the total flavonoid level was relatively higher in leaves than in roots or stems in both two kudzu genotypes (Figure 6A). Furthermore, total flavonoid content was higher in the roots of No. 1 than in No. 2, which was essentially contributed by puerarin as confirmed on high performance liquid chromatography (HPLC) in the present study (Figure 1). By contrast, total flavonoids were the lowest in stems in both kudzu genotypes (Figure 6A). Accordingly, the transcript level of *PlMYB1* determined by qPCR was relatively higher in leaves than in other tissues of both kudzu plants (Figure 6C). Notably, the transcript level of *PlMYB1* is exactly consistent with the accumulation levels of total flavonoids in various tissues. This result implied that *PlMYB1* was likely involved in the regulation of isoflavonoid biosynthesis. By contrast, the *PlMYB4-2* gene was highly expressed in the roots of both kudzu genotypes, with a very low level in stems and leaves (Figure 6E), implying a weaker association with total flavonoid accumulation.

It was revealed that *PlbHLH3-4* was expressed at the highest level in leaves of No. 1 kudzu genotype plants, but it accumulated at a low level in the leaves of the No. 2 kudzu genotype (Figure 6G), which is far more different from the total flavonoid accumulation pattern. Same as *PlbHLH3-4*, the transcript level of *PlWD40-1* was relatively high (more than 8-fold than in other tissues) in leaves of the No. 1 kudzu plant, but lower in other tissues of the No. 1 kudzu plant (Figure 6I, left), implying less correlation with total flavonoid accumulation.

### Key Transcription Factor Genes Were Consistently Associated With Puerarin Content in Cell Cultures

Puerarin is the predominant isoflavonoid compound in kudzu. It was revealed that the cell culture produced from the No. 1 kudzu genotype plant accumulated an evident amount of puerarin, and its content was not significantly affected by sugar or naphthyl acetic acid (NAA) concentration, or the presence of SA, MeJA, or light, but by the pH value of medium (Supplementary Figure 3). In comparison with the control pH value of 5.8, the puerarin content doubled when the pH value dropped to 4.8, whereas the puerarin content reduced about 6 folds when the pH value was increased to 6.8 (Figure 6B, right).

Quantitative PCR analyses showed that the transcript level of *PlMYB1* was relatively higher at a pH value of 4.8 than at 5.8 or 6.8 (Figure 6D). The transcript profile of *PlMYB1* is consistent with the accumulation levels of puerarin in cell cultures grown under different pH conditions. This result implied that *PlMYB1* is likely involved in the regulation of isoflavonoids, in particular puerarin biosynthesis. However, the expression level of *PlMYB4-2* was not affected by the pH value of the cell culture medium (Figure 6F, right), suggesting a weaker association with puerarin biosynthesis.

Similar to *PlMYB1*, the expression level of *PlbHLH3-4* in cell culture was increased at a pH value of 4.8 and decreased at a pH value of 6.8 as compared to the control at a pH value of 5.8 (Figure 6H). In addition, the transcript level of *PlWD40-1* was also higher at a pH value of 4.8 but lower at a pH value of 6.8 when compared with that of control at a pH value of 5.8 (Figure 6J, right).

Taken together, *PlMYB1*, *PlbHLH3-4*, and *PlWD40-1* were highly expressed in cell culture under a pH value of 4.8, and their expression patterns were highly correlated with levels of puerarin in cell cultures under various pH treatments, suggesting they might cooperate as an MBW complex to regulate the isoflavonoids, e.g., puerarin biosynthesis, under different pH treatments. Especially, the expression pattern of *PlMYB1* matched very well with the accumulation of puerarin, implying it is likely a key player in the MBW complex.

### Subcellular Localization of *PlMYB1*

**FIGURE 7:**
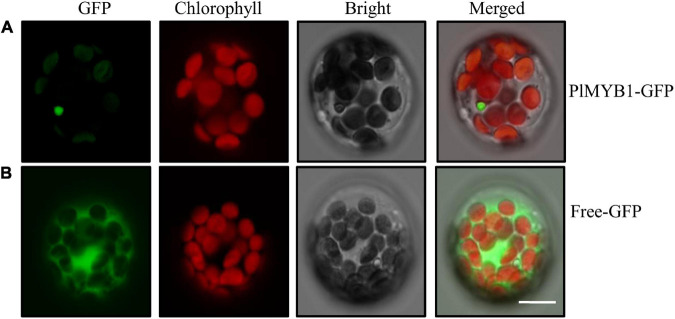
Subcellular localization of PlMYB1 protein. Subcellular localization assays of PlMYB1 fused with GFP in *Arabidopsis* protoplast. Fluorescence signals were visualized using confocal laser scanning microscopy. From left to right: green fluorescence, autofluorescence of chloroplast, bright field, and merged images of PlMYB1-GFP fusion protein **(A)**, and GFP **(B)**. Bar = 10μm.

To validate the function of the putative transcription factor in the regulation of the isoflavonoid pathway, the *PlMYB1* gene was successfully cloned for further characterization. The open reading frame (ORF) of *PlMYB1* was fused with green fluorescent protein (GFP) at the C-terminus and transferred into *Arabidopsis* leaf protoplasts. Green fluorescence signals for PlMYB1:GFP were detected in the nucleus (Figure 7A), which was evidently distinct from that of the control GFP in the cytosol (Figure 7B). This result indicated that *PlMYB1* is localized in the nucleus as a transcription factor to exert its function.

### *In vivo* Functional Characterization of *PlMYB1* in *Arabidopsis*

**FIGURE 8:**
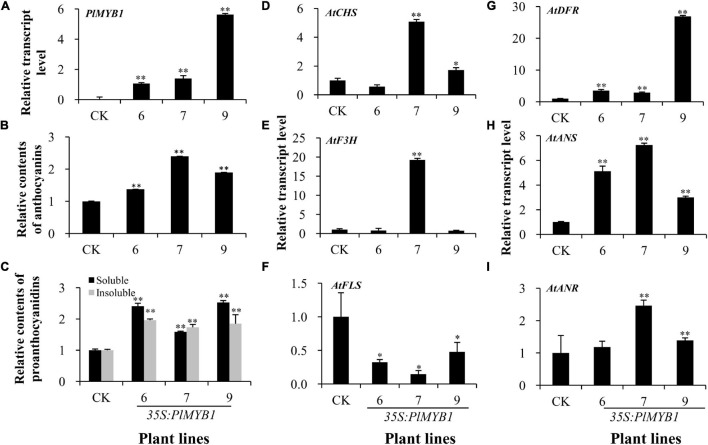
Over-expression of *PlMYB1* in *Arabidopsis*. **(A)** The relative transcript level of *PlMYB1* in the wild type of *Arabidopsis*. **(B,C)** The relative content of anthocyanins in rosette leaves **(B)** and proanthocyanidins in mature seeds **(C)** in the *PlMYB1* over-expressing homozygous *Arabidopsis*. Soluble proanthocyanidins were measured by using the DMACA-based method and insoluble proanthocyanidins were measured by using the butanol/HCl hydrolysis method. **(D–I)**. Relative transcript levels of *AtCHS*, *AtF3H*, *AtFLS*, *AtDFR*, *AtANS*, and *AtANR* in the rosette leaves of the transgenic and wild type *Arabidopsis* determined by qPCR analysis.

To further determine the regulatory function of *PlMYB1*, it was also over-expressed in the wild-type *Arabidopsis*. The expression levels of *PlMYB1* were confirmed by qPCR analysis in different lines (Figure 8A). Quantitative analysis revealed that anthocyanin levels increased by more than 0.4, 1.4, and 0.9-fold in rosette leaves of three transgenic lines as compared to the wild-type control (Figure 8B and Supplementary Figure 4A). In addition, soluble and insoluble proanthocyanidins in the mature seeds of these transgenic lines increased from 0.6- to 1.5-fold and 0.7- to 1-fold than the wild-type control, respectively (Figure 8C and Supplementary Figure 4B).

To further investigate the effects of *PlMYB1* on the expression of anthocyanin/proanthocyanidin pathway genes, the expression levels of several key pathway genes were determined in rosette leaves by qPCR analysis (Figures 8D–I). For the early pathway genes of *AtCHS* and *AtF3H*, they were both increased more significantly in the No. 7 transgenic line (Figures 8D,E). Especially, it showed that the expression levels of three later pathway genes *AtDFR*, *AtANS*, and *AtANR* genes were highly increased in these transgenic lines (Figures 8G–I). Notably, the expression level of *AtFLS* was significantly decreased in the transgenic lines than that in the wild-type control (Figure 8F). Taken together, these data indicated that the over-expression of *PlMYB1* activated the expression of anthocyanin/proanthocyanidin pathway genes, and consequently increased the accumulation of these compounds.

## Discussion

*Pueraria lobata* is a legume plant endemic to China, which is well known for its special accumulation of health-beneficial isoflavonoid compound of puerarin in the roots. In a previous study, we found that a kudzu genotype produces high puerarin content, while another genotype has low puerarin content in the roots (He et al., 2011; Figure 1). In the present study, we explored the transcriptomes of roots from two previously established kudzu plants. Transcriptome comparative analysis revealed several transcription factor genes were differentially expressed in two kudzu genotypes. Among them, the expression of *PlMYB1* showed a close correlation with the biosynthesis of puerarin, and it was able to significantly increase the accumulation of anthocyanins/proanthocyanidins in transgenic *Arabidopsis* through activating *AtDFR*, *AtANR*, and *AtANS* (Figure 8).

### *PlMYB1* Regulates Flavonoid Pathway in Kudzu and *Arabidopsis*

It is well known that flavonoid and isoflavonoid pathway genes were mainly regulated by the MBW complex comprising of MYB, bHLH, and WD40, and the regulatory function of MBW is conserved in most plants (Goodrich et al., 1992). In the MBW complex, MYB transcription factors are the major player, and they were classified into distinct groups. The MYBs in the Sg4 subgroup share the ERF-associated amphiphilic repression (EAR) motif core (Goodrich et al., 1992). The Sg4 subgroup members were involved in stress responses and plant evolution (Bedon et al., 2010), and also acted as a repressor factor of phenolic acid metabolism and lignin biosynthesis (Zhao et al., 2013). The MYBs members in *Arabidopsis* (AtMYB3, AtMYB4, AtMYB7, and AtMYB32) of the Sg4 subgroup are able to repress the biosynthesis of polyphenols by interacting with bHLH proteins (Zhao et al., 2013). AtMYB4 has been shown to be a transcriptional repressor involved in the inhibition of genes in the polyphenol biosynthetic pathway, such as the *Cinnamate 4-Hydroxylase* gene (*C4H*) (Jin et al., 2000).

Our study showed that two MYB members of the Sg4 subgroup were present in kudzu, namely *PlMYB4-1*, and *PlMYB4-2*. Among them, the expression pattern of *PlMYB4-2* was not consistent with the accumulation profiles of puerarin (Figures 6E,F), therefore, *PlMYB4-2* is unlikely related to puerarin accumulation in kudzu. By contrast, *PlMYB1* showed a high expression level in roots and leaves in both kudzu plants, which is completely consistent with the accumulation pattern of total flavonoid in various tissues of kudzu.

Moreover, a previous study demonstrated that pH condition is a key regulatory factor in flavonoid biosynthesis. It was found that treatment with a high medium pH value induced a dramatic decrease in the concentration of cyanidin in crabapple leaves (Zhang et al., 2014), whereas high medium pH values increased the content of flavones and flavonols (Zhang et al., 2014). Several MYB TFs have been suggested to be involved in the regulation of pH responses (Zhang et al., 2014). In our study, we found that low pH treatment increased puerarin content in kudzu cell cultures, and the transcript level of the *PlMYB1* gene was also accelerated (Figure 6). Particularly, *PlMYB1* was evidently increased under low pH value treatment and significantly decreased under high pH value treatment (Figure 6), which is consistent with the accumulation levels of puerarin under various pH value conditions. Therefore, *PlMYB1* possibly affected puerarin contents via regulating the transcript level of downstream key structural genes.

**FIGURE 9:**
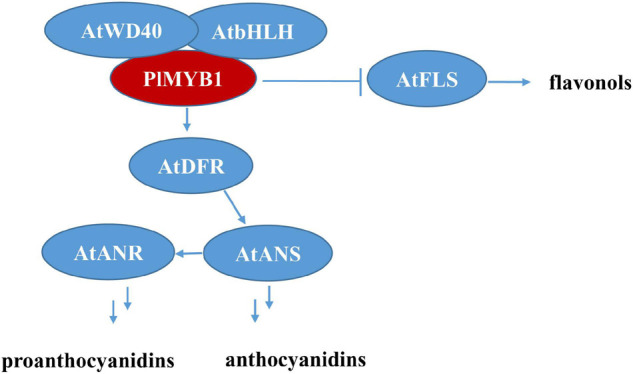
Predicted regulatory mechanism of *PlMYB1* in *Arabidopsis*. FLS, flavonol synthase; DFR, dihydroflavonol 4-reductase; ANS, anthocyanidin synthase; ANR, anthocyanidin reductase.

Furthermore, the ectopic over-expression of *PlMYB1* led to significant increases in the expression level of several pathway structural genes, e.g., *AtDFR*, *AtANS*, *AtANR*, Figure 8), and accordingly increased the content of anthocyanins and proanthocyanidins in *Arabidopsis*. These results demonstrated that PlMYB1 functioned as an activator for the anthocyanins/proanthocyanidins branch. Notably, the transcript level of *FLS* was reduced significantly (Figure 9), which might block the flavonol branch and switch the flux to the anthocyanidins/proanthocyanidins branch as illustrated in Figure 9, indicating *PlMYB* functioned as a repressor for the flavonol branch. Therefore, PlMYB1 acted dual role as activator and repressor in the flavonoid biosynthetic pathway in *Arabidopsis*, which is similar to VviMYB86 which oppositely regulates different flavonoid subpathways in grape berries (Cheng et al., 2020).

### *PlbHLH3-4* and *PlWD40-1* Co-expressed With *PlMYB1* Under Various pH Treatments

Most bHLH proteins can interact with R3 repeat domains of MYB proteins at the N-terminal acidic region to form the MYB–bHLH complex which frequently occurred in flavonoid biosynthetic pathways (Zhao et al., 2013). The bHLH members in subgroup IIIf were involved in anthocyanin biosynthesis (Spelt et al., 2000), seed coat differentiation, trichome formation, and root hair formation (Nesi et al., 2001). In addition, *bHLH* genes in subfamily 2 were found to generally respond to wounds, insects, drought, oxidative stress, jasmonic acid, abscisic acid, and chitin, but they also are able to regulate anthocyanin metabolism (Carretero-Paulet et al., 2010). AtbHLH13 is a member of subfamily 2 (Heim et al., 2003) and subgroup IIId (Song et al., 2013) as well.

In this study, three *bHLH* genes in kudzu, namely *PlbHLH3-2*, *PlbHLH3-3*, *PlbHLH3-4*, belong to the subfamily 2. They showed high similarities to *AtbHLH13*, with 63%, 65%, and 48% identity at amino acid level, respectively. The expression patterns of the *PlbHLH3-4* gene were well consistent with total flavonoid content in the roots and leaves of high puerarin kudzu, but were inconsistent with that in low puerarin kudzu, implying *PlbHLH3-4* might be involved in isoflavonoid biosynthesis but is not the determinant factor in kudzu. Interestingly, the expression of the *PlbHLH3-4* gene was evidently increased under low pH medium and significantly decreased under high pH conditions, which is completely consistent with the accumulation of puerarin, suggesting *PlbHLH3-4* was possibly responsible for the biosynthesis regulation of puerarin under various pH conditions. Moreover, PlbHLH3-4 was found to be localized in the nucleus as PlMYB1 at the subcellular level (Supplementary Figure 5), implying that it is likely to interact with PlMYB1 in the nucleus as a major regulator for the flavonoid pathway.

Except for *bHLH*, *Arabidopsis WD40* like *AtTTG1* also plays a key role in the regulation of flavonoid biosynthesis. *AtTTG1* plays an important part in the regulation of *AtDFR*, *AtANS*, and *AtANR* by interacting with bHLH transcription factors (AtGL3, AtEGL3, or AtTT8) and *MYB* transcription factors (AtPAP1, AtPAP2, AtMYB113, or AtMYB114) in the MBW complex. This ternary MBW complex was known for controlling proanthocyanidin accumulation in seeds and anthocyanin accumulation in leaves (Walker et al., 1999; Payne et al., 2000; Baudry et al., 2004; Hichri et al., 2011). MdTTG1 identified from *M. domestica* was capable of fully replacing AtTTG1 to activate *AtBAN* promoter in cooperation with AtTT2 and AtTT8 in a co-transfection system (An et al., 2012). In *M. truncatula*, the deficiency of *MtWD40-1* expression strongly suppressed the expression of flavonoid structural genes and thus blocked the accumulation of a range of flavonoid compounds (Pang et al., 2009).

In the present study, kudzu PlWD40-1 showed high sequence similarity with MdTTG1 and MtWD40-1 (Figure 5). The expression profiles of the *PlWD40-1* gene were less correlated with the accumulation of total flavonoids in either high or low puerarin kudzu plants. However, the expression of the *PlWD40-1* gene was highly consistent with the puerarin accumulation under various pH treatments, suggesting *PlWD40-1* possibly involved in the biosynthesis regulation of puerarin under various pH conditions.

As *PlbHLH3-4* and *PlWD40-1* showed very high similarity with *AtTT8* and *AtTTG1* of *Arabidopsis*, and they displayed similar transcript profiles to *PlMYB1* under various pH treatments (Figure 6), thus PlMYB1, PlbHLH3-4, and PlWD40-1 might form an MBW complex to regulate the accumulation of isoflavonoids in kudzu. In particular, *PlbHLH3-4* and *PlWD40-1* possibly play key roles in the isoflavonoid biosynthesis under various stresses like pH stimuli.

In summary, PlMYB1 acted as a potent transcript factor to regulate the production of various flavonoids/isoflavonoids. The expression profile of the *PlMYB1* gene was significantly consistent with the total flavonoid level in kudzu. Furthermore, expression levels of *PlMYB1* together with *PlbHLH3*-4 and *PlWD40-1* were consistent with the puerarin content under various pH treatments. Therefore, it is reasonable to speculate that PlMYB1, PlbHLH3-4, and PlWD40-1 should cooperate together to finely tune the production of various isoflavonoids in kudzu. Overexpression of *PlMYB1* induced a significant increase of anthocyanins/proanthocyanidins as well as related biosynthesis pathway genes. Our investigation could shed some light on the regulation network of isoflavonoid biosynthesis in kudzu and provide a potential gene target for the bioengineering of particular flavonoids in plants.

## Materials and Methods

### Transcriptome Sequencing and *de novo* Assembly

The roots of the two previously reported kudzu plants were propagated and collected separately at 7, 14, and 21 days after rooting. Kudzu plant of No. 1 accumulates puerarin, but No. 2 does not (He et al., 2011). Root materials were immediately frozen in liquid nitrogen (LN) and stored at –80°C prior to further analysis. Total RNAs were extracted with Tri-reagent according to the protocol of the manufacturer (Invitrogen, Waltham, MA, United States), followed by cleaning and purification with the DNase I. Equal amount of RNA from 7-, 14-, and 21-day-old root samples from Nos. 1 and 2 kudzu plant were pooled together, respectively, for sequencing with a biological triplicate. Poly(A) mRNA was purified from total RNA with polyoligo d(T) attached magnetic beads and then broken into short fragments, which were used as templates for double-stranded cDNA synthesis using random hexamer primers. The double-stranded cDNAs were purified, connected with sequencing adapters, and were separated by gel electrophoresis (Liuyi, Beijing, China). The purified double-stranded cDNAs with an average insert size of 400 bp were sequenced by the Illumina sequencing platform (San Diego, CA, United States). Reads were then assembled into contigs using Trinity software.

### Functional Annotation of Unigenes

Functional annotations of the unigenes were performed by alignment of the assembly with unigenes against the NCBI Nr, SwissProt (UniProt Consortium, Switzerland), KOG^1^, and Kyoto Encyclopedia of Genes and Genomes (KEGG) databases^2^ using BLASTX (*E*-value < 10^–5^). The proteins from the Nr database with the highest hits to the unigenes were used to assign functional annotations. GoPipe software was used to analyze the GO annotations and GO functional classifications (Chen et al., 2005) (IGDB-CAS, Beijing, China).

The expression levels of unigenes were calculated using the reads per kb per million reads (RPKM) method, which eliminates the influence of different gene lengths and sequencing discrepancies (Mortazavi et al., 2008). Thus, this method can be used directly for comparing the differences in gene expression levels between two types of *P. lobata* roots. The fold-change in each gene expression in the two samples was calculated from the ratio of the RPKMs. In this study, differentially expressed genes (DEGs) were screened with an absolute value of log_2_ ratio > 2 and a threshold of false discovery rate (FDR) value lower than 0.005 (Wang et al., 2010). The identified DEGs were mapped to each term of the GO database^3^ and we calculated the gene numbers in each GO term. In addition, DEGs were also used in pathway enrichment analysis by calculating the gene numbers which mapped to KEGG in each pathway (Kanehisa et al., 2010).

### Sequence Analysis

Alignments were performed using Clustal W algorithm-based AlignX module (UCD, Dublin, Ireland). The rooted trees were constructed using the ML method with MEGA X Software (Kumar et al., 2018). Tree nodes were evaluated by bootstrap analysis for 1,000 replicates (pairwise deletion, uniform rates, and Poisson correction options). Evolutionary distances were computed using the *p*-distance method and expressed in units of amino acid differences per site. All positions containing gaps and missing data were eliminated prior to the construction of phylogenetic trees.

### Cloning and Expression of Candidate Genes in *P. lobata*

Putative transcription factor genes were cloned from the roots of *P. lobata* No. 1. First-strand cDNA was synthesized from total RNAs of roots using FastQuant RT Kit (TIANGEN, China). The primary PCR was performed using cDNA from the roots and the PCR conditions were as following: 5 min of initial denaturation at 94°C, followed by 94°C for 30 s, 54°C for 30 s, and 72°C for 1 min in a 35-cycle reaction, and a final elongation step of 72°C for 7 min. Primers sequences were listed in Supplementary Table 6.

Quantitative PCRs were performed on a Bio-Rad CFX96^TM^ Real-time PCR System using SYBR Real Master Mix (Kangwei, China). The *Arabidopsis pp2A* gene (accession No. U39568) was used as an internal reference gene for the calculation of relative transcript levels. Primers used for genes from kudzu were listed in Supplementary Table 6, and primers for genes from *Arabidopsis* were the same as in our previous study (Su et al., 2020). Each reaction (20 μl) contained 1 μM each primer, 1 μl cDNA (1:10 diluted), 7 μl RNase-free H_2_O, and 10 μl PCR buffer of SYBR Real Master Mix. Thermal cycling conditions were as follows: pre-incubation at 95°C for 10 min, followed by 95°C for 20 s, 60°C for 30 s, and 72°C for 20 s for 40 cycles. Data were calculated from three biological replicates, and each biological replicate was examined in triplicate.

### Subcellular Localization Analysis of *Candidate genes*

The coding region of transcription factor genes was amplified with prime pairs containing *Sal* I and *Bam* HI restriction sites, respectively (Supplementary Table 6). The resulting amplification product was digested and ligated to the same enzyme digested destination vector PJIT163hGFP. After confirmation by sequencing, the recombinant constructs were used in *Arabidopsis* protoplast transformation as described previously (Sheen, 2002). GFP fluorescence in *Arabidopsis* protoplast cells was detected by laser scanning confocal microscopy using Leica TCS SP5 (Wetzlar, Germany). The emission was collected for GFP from 500 to 560 nm, and for the chlorophyll from 605 to 700 nm.

### Establishment and Treatment of *P. lobata* Cell Suspension Culture

Stems of kudzu plant No. 1 were surface sterilized in 75% (v/v) ethanol for 1–2 min, followed by three washes in sterile distilled water, 15 min in 10% hydrogen peroxide, and another three washes in distilled water. Then the axenic stems were cut into pieces in 1 cm length and planted on MS basal medium (pH = 5.8) with 3% sucrose, 0.8% agarose, 1 mg/L L-NAA, and 2 mg/L 6-benzylaminopurine (BA). Two weeks later, the emerged calli were transformed to B5 liquid medium (pH = 5.8), supplemented with 1 mg/L 2, 4-dichlorophenoxyacetic acid (2, 4-D), 1 mg/L NAA, 0.5 mg/L kinetin (KT), and 1% casein hydrolysate. Calli were then sub-cultured every week and incubated at 25 ± 2°C with a 16/8 h photoperiod. Cell culture was incubated in a flask on a rotary shaker (110–130 rpm) under the same photoperiod at 25°C. After about 1 month, soft, loose, and pale green calli were obtained. Once established, cultures were periodically sub-cultured into 100 ml flasks by transferring 15 ml 7-day-old cells into 40 ml fresh B5 liquid medium for treatment. For the treatment with salicylic acid (SA, 0.1 mg/L) and methyl jasmonate (MeJA, 1 mg/L), the cell cultures were collected at time points of 0, 2, 4, 8, 16, 24, 48, and 72 h. The fresh samples were collected and used for flavonoid analysis on HPLC with a triplicate.

### Generation of Transgenic *Arabidopsis* Plants

To produce *PlMYB1*-overexpressing *Arabidopsis* plants, the 651-bp CDS (coding sequence) fragment was amplified by PCR, and then cloned into the binary vector pCXSN at the *Xcm* I site for gene over-expression in the wild-type *A. thaliana*. The resulting sequenced pCXSN-PlMYB1 construct was transformed into *Agrobacterium* strain GV3101 and used to generate transgenic *A. thaliana* plants by using the inflorescence dip method (Clough and Bent, 1998). The transgenic *A. thaliana* plants were screened on mass spectrometry (MS) medium supplied with hygromycin (30 mg/L). The 30-day-old rosette leaves and seeds of T_3_ generation homozygous plants were collected for further analyses.

### Analyses of Flavonoid Compounds

For the extraction and quantification of total flavonoids, plant materials were all ground into powder and freeze-dried. Ten milligram dry powders were extracted by sonication for 30 min with 500 μl of 80% methanol in biological triplicates. After an additional overnight extraction at 4°C, the extracts were centrifuged at 12,000 rpm for 10 min. Deionized water amounting to 400 μl and 30 μl 5% NaNO_2_ was added to every 100 μl supernatant, followed by the addition of 30 μl 10% AlCl_3_ after 5 min. Later, 200 μl of 1 M NaOH were added 10 min, followed by the addition of 240 μl deionized water to make the final volume to 1 ml. The absorbance at 510 nm was measured using a spectrophotometer with quercetin as standard (BIO-RAD, CA, United States).

The above methanolic extracts were also applied for the identification and quantification of isoflavonoids on HPLC. The analyses were carried out using an Agilent 1260 chromatographic system (Santa Clara, CA, United States) equipped with a quaternary pump, an autosampler, a photodiode array detector, and Eclipse XDB-C18 reverse-phase column (4.6 mm × 250 mm, 5 μm). Flavonoids were separated with a linear eluting gradient (5–70% solvent B over 30 min) with solvent A (0.1% formic acid in water) and solvent B (0.1% formic acid in acetonitrile) at a flow rate of 1 ml/min and detected at 254 nm. The synthetic standard used in this study were all purchased from Sigma-Aldrich (Darmstadt, Germany).

Anthocyanins were extracted in acidified MeOH (0.1% HCl) overnight in the dark at 4°C, followed by sonication for 30 min. After centrifugation at 12,000 rpm for 10 min, the supernatant was mixed with the same volume of water and extracted with chloroform. The supernatant was then measured at 530 nm using a spectrophotometer. Three independent replicates were collected for each infiltration.

Proanthocyanidins were extracted three times with 70% acetone (0.5% acetic acid). Pooled supernatants were then extracted with chloroform and hexane. The soluble and insoluble proanthocyanidins were determined by dimethylaminocinnamaldehyde (DMACA) staining and butanol-hydrochloric acid (HCl) hydrolysis, respectively, as previously reported (Pang et al., 2007).

## Data Availability Statement

The datasets presented in this study can be found in online repositories. The RNA-Seq data presented in the study are deposited in the NCBI SRA repository with accession number PRJNA747842. The sequences of *PlMYB1*, *PlbHLH3-4*, and *PlWD40-1* genes are deposited at Genbank with accession Nos. KR698796, KT236099, and AKR04124.1, respectively.

## Author Contributions

GS and YP: conceptualization and funding acquisition. GS, RW, and YP: methodology. GS: software and data analysis. RW and YX: investigation. YP: resources, writing-review and editing, and supervision. GS and RW: writing-original draft preparation. All authors have read and agreed to the published version of the manuscript.

## Conflict of Interest

The authors declare that the research was conducted in the absence of any commercial or financial relationships that could be construed as a potential conflict of interest.

## Publisher’s Note

All claims expressed in this article are solely those of the authors and do not necessarily represent those of their affiliated organizations, or those of the publisher, the editors and the reviewers. Any product that may be evaluated in this article, or claim that may be made by its manufacturer, is not guaranteed or endorsed by the publisher.

## Footnotes

1
http://www.ncbi.nlm.nih.gov/COG

2
http://www.genome.jp/kegg/

3
http://www.geneontology.org/
