# Supplementary material for: Identification of Transcription Factor Genes and Functional Characterization of PlMYB1 From Pueraria lobata
Source: Front Plant Sci. 2021 Oct 8;12:743518. doi: 10.3389/fpls.2021.743518 (PMC8531098; doi:10.3389/fpls.2021.743518)
Supplement: Supplementary file 1 [file Data_Sheet_1.docx]

**Supporting Information**

**Figure S1.** The biosynthetic pathway of isoflavonoids in kudzu plants. PAL, phenylalanine ammonialyase; C4H, cinnamate 4-hydroxylase; 4CL, 4-coumarate-CoA ligase; CHS, chalcone synthase; CHI, chalcone isomerase; CHR, chalcone reductase; IFS, isoflavone synthase; HID, trihydroxyisoflavanone dehydratase; IOMT, isoflavone O-methyltransferase; UGT, UDP-glycosyltransferase.

**Figure S2.** Statistic analysis of the kudzu root transcriptome data. (a). Length distribution of unigenes in transcriptome of kudzu root. (b) Venn diagram showing shared genes up-regulated and down-regulated in the two kudzu transcriptome.

**Figure S3.** The content of puerarin in cell culture of *P. lobata* under different culture conditions. (a) The conditions in CK medium was as followed, sugar (30g/L), 2,4-D (1mg/L), NAA (1mg/L), KT (0.5mg/L), pH5.8, and under light. (b-c) The cell cultures were treated with 0.1mg/L SA and 1mg/L MeJA at 2, 4, 8, 16, 24, 48, 72 hours. The samples were analyzed on HPLC with a triplicate.

**Figure S4.** Over-expression of *PlMYB1* in wild type *Arabidopsis* plants. (a) Anthocyanin phenotype of the *Arabidopsis* over-expression line 9 and the wild type control (CK) and red arrowhead indicated the accumulation of anthocyanin in the bottom of leaves. (b) Seed phenotype of the *Arabidopsis* over-expression line 9 and the wild type control (CK) before (upper panels) and after DMACA staining (lower panels).

**Figure S5. Subcellular localization of PlHLH3-4 protein.** Subcellular localization assays of PlHLH3-4 fused with GFP in *Arabidopsis* protoplast. Fluorescence signals were visualized using confocal laser scanning microscopy. From left to right: green fluorescence, autofluorescence of chloroplast, bright field, and merged images of PlHLH3-4-GFP fusion protein (upper panel) and GFP (lower panel). Bar=10μm.
